# Cell-Specific Suppression of 4-Coumarate-CoA Ligase Gene Reveals Differential Effect of Lignin on Cell Physiological Function in *Populus*

**DOI:** 10.3389/fpls.2020.589729

**Published:** 2020-11-17

**Authors:** Shumin Cao, Cheng Huang, Laifu Luo, Shuai Zheng, Yu Zhong, Jiayan Sun, Jinshan Gui, Laigeng Li

**Affiliations:** ^1^National Key Laboratory of Plant Molecular Genetics, CAS Center for Excellence in Molecular Plant Sciences, Shanghai Institute of Plant Physiology and Ecology, Chinese Academy of Sciences, Shanghai, China; ^2^University of the Chinese Academy of Sciences, Beijing, China

**Keywords:** 4-coumarate-CoA ligase gene, lignin, vessel, xylem, *Populus*, fiber cell

## Abstract

Lignin is a main component of the secondary cell wall in vessels and fibers of xylem tissue. However, the significance of lignin in cell physiology during plant growth is unclear. In this study, we generated lignin-modified *Populus* via cell-specific downregulation of the 4-coumarate-CoA ligase gene (*4CL*). The transgenic plants with selective lignin modification in vessel elements or fiber cells allowed us to investigate how lignin affects the physiology of vessel or fiber cells in relation to plant growth. Results showed that vessel-specific suppression of lignin biosynthesis resulted in deformed vessels and normal fibers, while fiber-specific suppression of lignin biosynthesis led to less-lignified fibers and normal vessels. Further analyses revealed that the efficiency of long distance water transport was severely affected in transgenics with vessel-specific lignin modification, while minimal effect was detected in transgenics with fiber-specific lignin modification. Vessel-specific lignin reduction led to high susceptibility to drought stress and poor growth in field, likely due to vessel defects in long distance transport of water. The distinct physiological significance of lignin in different cell types provides insights into the selective modification of lignin for improvement of lignocellulosic biomass utilization.

## Introduction

Lignin emerged along with the evolution of vascular plants. It is believed that the advent of lignin enabled early tracheophytes to stand upright and to expand in body size (Weng and Chapple, 2010). In conventional angiosperms, lignin is deposited mainly in vascular tissue, such as in vessel cells and fibers in xylem. Vessel cells, which facilitate a channel for long distance transport, play a different role in supporting plant growth from fibers, which are mainly responsible for providing mechanical support.

In the cell walls of vessels and fibers, lignin, cellulose, and hemicelluloses are woven together to form rigid walls, which provide characteristic structure for cells to function properly. Genetic evidence shows that defective biosynthesis of lignin, cellulose, or hemicelluloses results in wall damage in xylem tissue and abnormal plant growth (Turner and Somerville, 1997; Zhong et al., 1998; Taylor et al., 1999; Franke et al., 2002; Persson et al., 2007; Voelker et al., 2010; Xi et al., 2017; Gui et al., 2019, 2020). Plant cell walls store a majority of photosynthesis-accumulated energy and carbon and are the most abundant resource potentially for production of renewable energy and biomaterials (Weng et al., 2008). The lignin-impregnated walls are recalcitrant to hydrolysis and negatively affect its utilization for producing biofuels and bio-based chemicals (Kumar et al., 2016; Nishimura et al., 2018). Bioengineering of cell wall lignin to improve the utilization of cell wall biomass has attracted immense interest (Li et al., 2003; Petersen et al., 2012; Liu et al., 2014; Mottiar et al., 2016; De Meester et al., 2018). However, the modification of lignin biosynthesis in xylem is often associated with growth defects (Li et al., 2010; Voelker et al., 2010; Bonawitz and Chapple, 2013; Gui et al., 2019). We sought to examine the correlation between wall components and cell functionality in order to develop new strategies to modify the cell wall without impeding plant growth.

In Arabidopsis, *c4h* mutant show retarded growth, while vessel-specific reintroduction of *C4H* gene was able to restore the dwarfed phenotype (Yang et al., 2013). Similarly, vessel-specific expression of *CCR1* in the dwarfed *ccr1* mutant restored vessel integrity (De Meester et al., 2018). Our recent study shows that cell type-specific modification of lignin biosynthesis in xylem vessels and fibers displayed different effects on growth in *Populus* (Gui et al., 2020). Thus, one remaining question to be answered is how lignin biosynthesis affects cell functionality in correlation to plant growth.

In this study, we employed a strategy to regulate lignin biosynthesis in specific cells in *Populus* xylem. Expression of the lignin-specific 4-coumarate-CoA ligase gene (*4CL*) was suppressed in *Populus* under the control of either a vessel-specific or a fiber-specific promoter. The transgenics, which were modified with different lignin biosyntheses in xylem vessels and fibers were analyzed for their growth, efficiency of long distance water transportation, mechanical strength, and response to drought stress. This study reveals distinct physiological significance of lignin in different cells as related to plant growth.

## Results

### Specific Downregulation of *4CL* Expression in Fibers and Vessels

Previous studies have shown that downregulation of *4CL* expression under the control of a constitutive promoter or a tissue-specific promoter led to the reduction in lignin content in xylem tissue (Kajita et al., 1996; Lee et al., 1997; Hu et al., 1999; Li et al., 2003; Gui et al., 2011). To investigate how lignin biosynthesis can be modified through cell-specific suppression of *4CL*, we carried out suppression of the lignin-specific *4CL* in the fibers and vessels of *Populus* xylem, respectively. We employed a fiber cell-specific promoter from the *WND1B* (*Potri.001G448400*) gene, which is specifically expressed in fibers (Zhong et al., 2010; Zhao et al., 2014), to downregulate *4CL* expression. The *WND1B* promoter (designated as *WND1Bp*) was cloned and combined to a 35S minimal promoter to construct a synthetic promoter *WND1Bp-35Smini*. After being fused to a β*-glucuronidase* (*GUS*) gene (*WND1Bp-35Smini-GUS*) (Supplementary Figure S1A), its fiber-specific activity was verified in *Populus*. As shown in Supplementary Figure S1B, the high GUS activity was detected in xylem fibers. Immunolocalization using GUS-specific antibodies further confirmed that the *WND1Bp-35Smini-GUS* signal was detected in fiber cells (Supplementary Figures S1C,D). For vessel-specific suppression, a promoter from *Populus* XYLEM CYSTEINE PROTEASE1 (*XCP1*; *Potri.004G207600*) was cloned and combined with a 35S minimal promoter to generate *XCP1p-35Smini*, which has been verified for its vessel-specific activity (Gui et al., 2020), was used for downregulation of *4CL* expression in xylem vessels.

**FIGURE 1:**
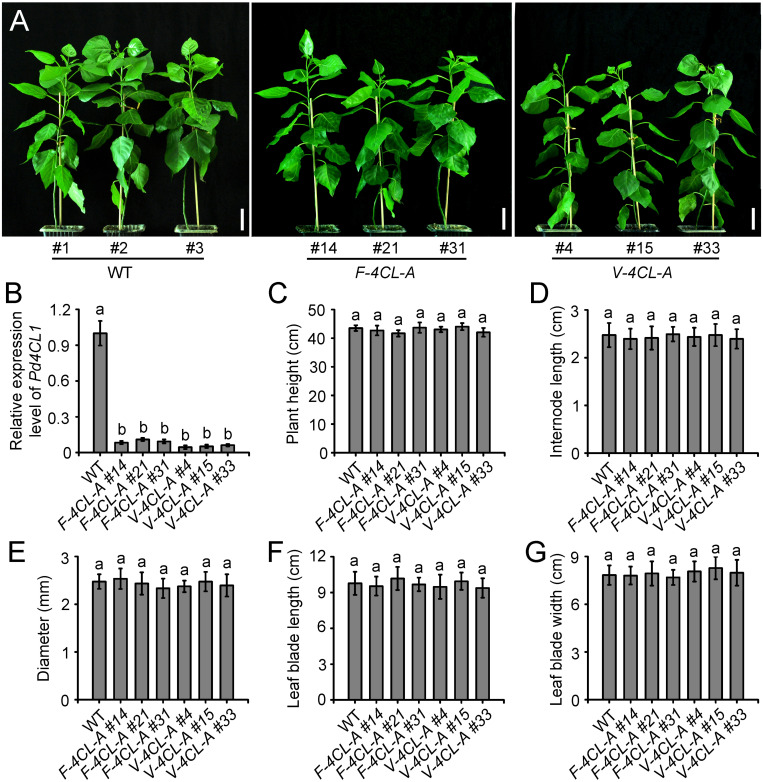
Phenotypes of the vessel-specific and fiber-specific down-regulation of *4CL1* expression in *Populus*. **(A)** Phenotypes of the transgenics through fiber-specific (*F-4CL-A*) and vessel-specific (*V-4CL-A*) downregulation of *4CL1* expression grown in phytotron at 2 months old. Scale bar, 5 cm. **(B)** Expression of *4CL1* gene in *4CL1* antisense transgenic plants. Gene expression in the control was set as 1. Results are means ± SE of three biological replicates. **(C–G)** Plant height **(C)**, internode length **(D)**, stem diameter **(E)**, leaf blade length **(F)**, and leaf blade width **(G)** of the transgenic and control plants were measured at 2 months old. Plant height and stem diameter: means ± SE of 5 clonally propagated plants; the other parameters: means ± SE of 20 internodes from 5 plants. Different lowercase letters in **(B)** indicate significant differences at *p* < 0.01 by ANOVA.

The lignin-specific *4CL1* (*Potri.001G036900*) gene (Li et al., 2003) was used for lignin biosynthesis modification. The *4CL1* was downregulated through antisense suppression under the control of the two synthetic promoters, respectively. The constructs *WND1Bp-35Smini-4CL1AS* and *XCP1P-35Smini-4CL1AS* were transferred to *Populus*, and the transgenic plants that harbored *WND1Bp-35Smini-4CL1AS* were designated as *F-4CL-A* and *XCP1P-35Smini-4CL1AS* as *V-4CL-A*, respectively. Forty-three independent *F-4CL-A* transgenic lines were identified and three representative transgenic lines, *F-4CL-A #14*, *F-4CL-A #21*, and *F-4CL-A #31* were clonally propagated for detailed morphological characterization (Figure 1A). Meanwhile, forty-six independent *V-4CL-A* transgenic lines were generated. Three representative transgenic lines, *V-4CL-A #4*, *V-4CL-A #15*, and *V-4CL-A #33*, were clonally propagated and used for morphological characterization (Figure 1A). In both *F-4CL-A* and *V-4CL-A* transgenics, *4CL1* expression was significantly suppressed compared to the WT (Figure 1B). During early growth in a phytotron, *F-4CL-A* and *V-4CL-A* displayed a similar growth in plant height, internode length, stem diameter, leaf blade length, and width (Figures 1C–G). The cell-specific downregulation of *4CL* expression in *Populus* showed minimal effect on the growth in phytotron.

**FIGURE 2:**
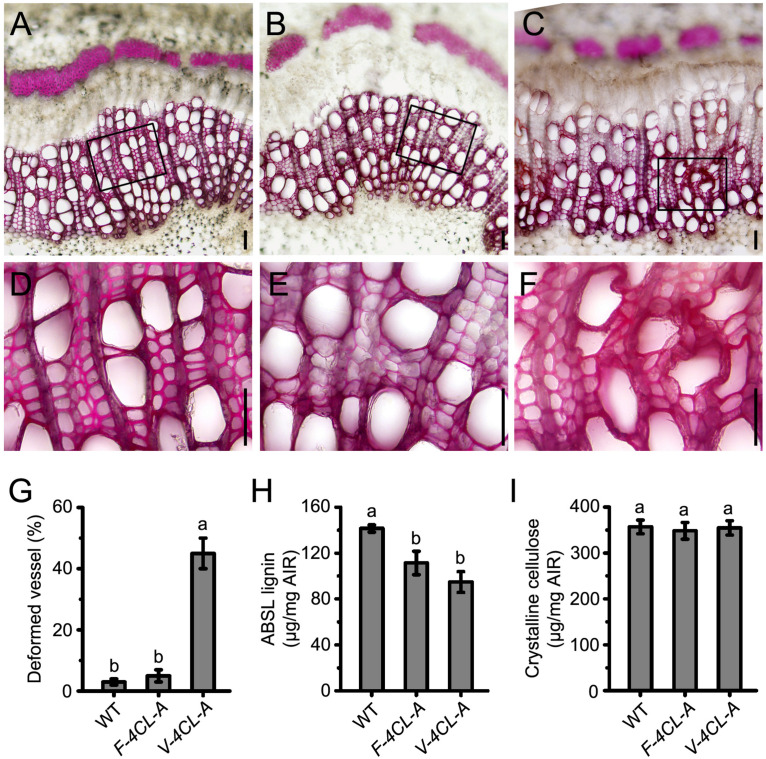
Vessel-specific and fiber-specific downregulation of *4CL1* expression resulted in reduction of lignin biosynthesis in vessels and fibers, respectively. **(A–F)** Cross sections (11th internode) of the wild-type control **(A)**, *F-4CL-A* **(B)**, and *V-4CL-A* **(C)** stained for lignin using phloroglucinol-HCl. Close-up images of the control **(D)**, *F-4CL-A* **(E)**, and *V-4CL-A* **(F)** indicated with a rectangle in the upper panel **(A–C)**. Scale bar, 50 μm. **(G)** The percentage of deformed xylem vessels in the WT, *F-4CL-A*, and *V-4CL-A* trees. Five biological replicates from two independent lines were examined for the count. **(H,I)** Lignin content **(H)** and crystalline cellulose content **(I)** in the stems of the 2-month-old plants. Results are means ± SE of three biological replicates of three independent lines. ABSL, acetyl bromide-soluble lignin. Different lowercase letters in **(G,H)** indicate significant differences at *p* < 0.01 by ANOVA.

Then we examined the lignin content and xylem structure of the *4CL1*-suppressed transgenics. In the *F-4CL-A* transgenics, lignin deposition was reduced in fiber cell walls with no apparent changes in vessel walls (Figures 2A,B,D,E). In contrast, the *V-4CL-A* transgenics developed deformed and less-lignified vessels, but lignin deposition in fiber cell walls was normal (Figures 2A,C,D,F). More than 40% vessels showed wall-deformed structure in the *V-4CL-A* transgenics (Figure 2G). Determination of lignin content in the transgenic plants showed that lignin was reduced by 21% in the *F-4CL-A* transgenics and by 26% in the *V-4CL-A* transgenics (Figure 2H). Meanwhile, the content of crystalline cellulose had minimal change in the transgenics (Figure 2I). The results indicate that lignin deposition was reduced by cell-specific downregulation of *4CL* expression in *Populus*.

### Vessel-Specific Suppression of Lignin Biosynthesis Affected the Functionality of Long Distance Transportation System

**FIGURE 3:**
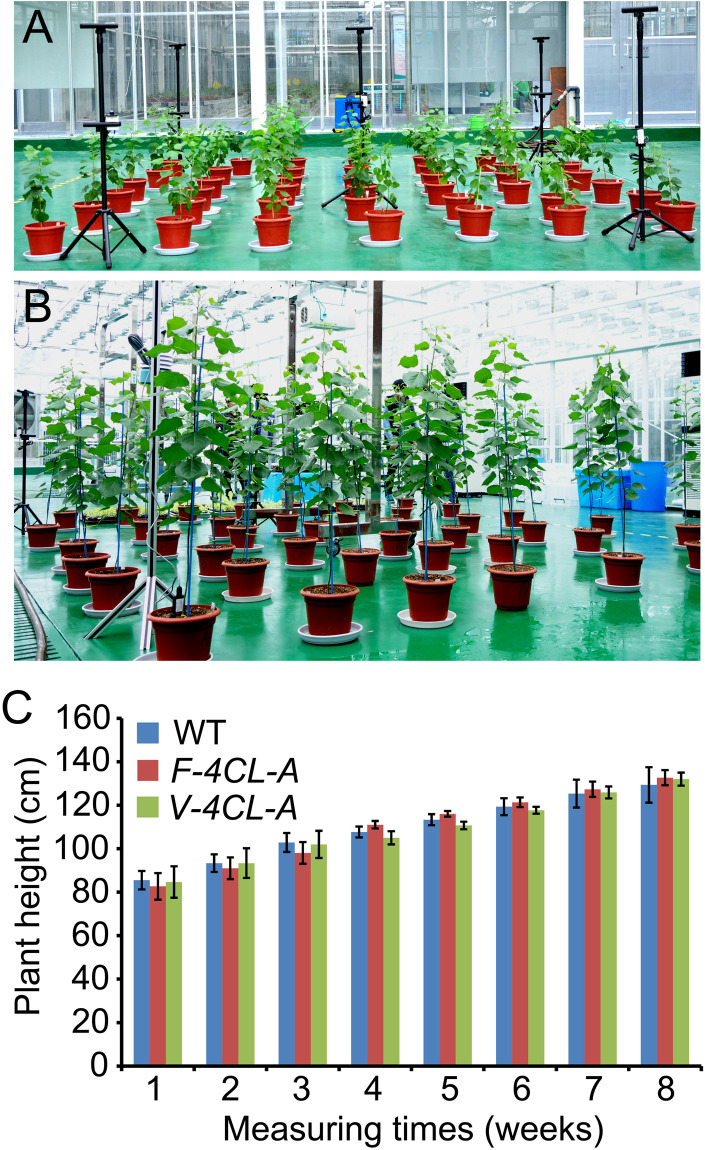
Continuous phenotyping of the transgenic *Populus* under greenhouse conditions. **(A,B)** The transgenic *Populus* through vessel-specific or fiber-specific downregulation of *4CL1* expression were clonally propagated (12 copes for each transgenic) and grown in a greenhouse at 2 months old **(A)** and at 4 months old **(B)**. **(C)** Plant height was recorded in a period of a continuous 2 months. Results are means ± SE of 12 clonally propagated plants from 3 independent lines.

In order to evaluate how cell-specific regulation of lignin affects plant growth, the *F-4CL-A* and *V-4CL-A* transgenics were propagated through micro-cutting and grown in a greenhouse (Figures 3A,B). The plant growth and morphology were recorded through a phenotyping system (LemnaTec 3D Scanalyzer) (Supplementary Figure S2). In greenhouse condition, the leaf size and shape of the *F-4CL-A* and *V-4CL-A* transgenics were similar to those of the WT. Plant growth in height did not show a difference between the *F-4CL-A*, *V-4CL-A* transgenics, and WT plants during a continuous observation of 8 weeks (Figure 3C). Then, we further measured leaf stomata conductance of these plants (Supplementary Figure S3). Stomata conductance in *F-4CL-A* transgenics and *V-4CL-A* transgenics were similar to that of the WT (Supplementary Figure S4). These results suggest that a fiber (*F-4CL-A*) or a vessel (*V-4CL-A*) cell-specific downregulation of *4CL1* expression in *Populus* did not show a significant effect on growth under the greenhouse condition.

**FIGURE 4:**
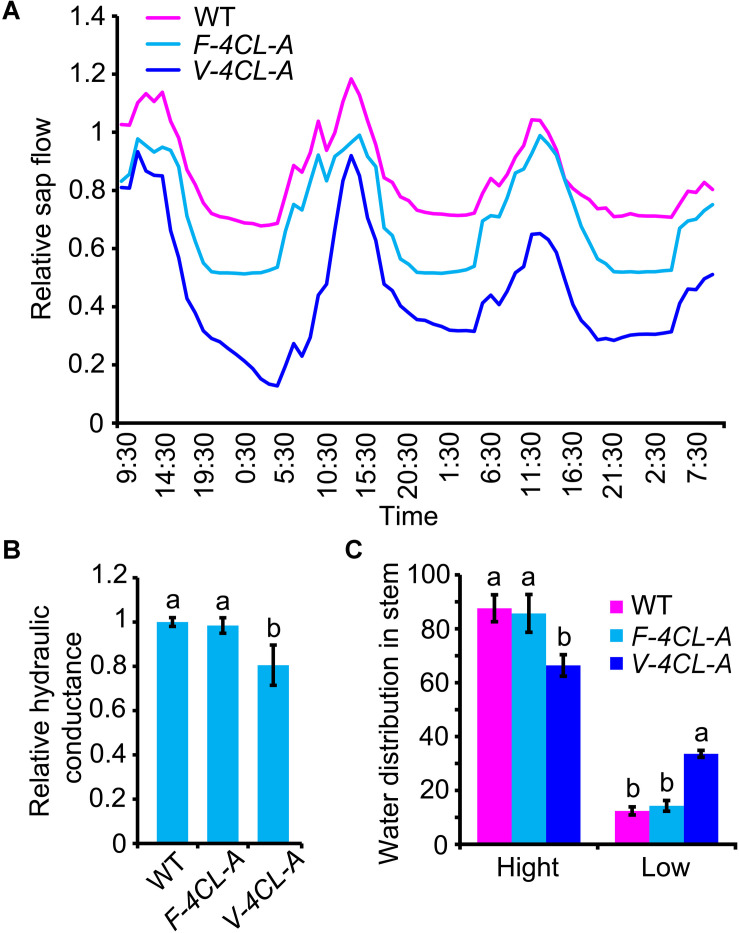
Vessel-specific downregulation of *4CL1* expression affected stem sap flow and water conductance. **(A)** Stem sap flow was recorded continuously for 3 days, and three biological replicates were carried out. One representative graph is shown. **(B)** Hydraulic conductance was measured using a High Pressure Flowmeter (HPFM). The hydraulic conductance in control plants was set as 1. Relative hydraulic conductance: means ± SE of 12 clonally propagated plants from 3 independent lines. **(C)** Water content distribution was estimated based on the near-infrared chemometric imaging. The distribution of low water content and high water content was calculated based on the stem near-infrared (NIR) images. The values represent means ± SE of 12 clonally propagated plants from 3 independent lines. Different lowercase letters in **(B,C)** indicate significant differences at *p* < 0.01 by ANOVA.

Vessels and fibers in xylem constitute a system to facilitate long distance transportation of water and to reinforce mechanical support for sustaining plant growth. To examine whether the cell-specific modification of lignin biosynthesis in xylem affects the system function in water transportation, we examined stem sap flow, stem water distribution, and stem hydraulic conductance in the transgenic plants. Stem flow sensors were used to continuously monitor the stem flow within 72 h (Supplementary Figure S5). The stem sap flow displayed a cycle of 24-h period with a peak in daytime and a valley in nighttime (Figure 4A). F-4CL-A, V-4CL-A transgenics, and WT plants showed a similar 24-h cycle in the stem sap flow. However, the rate of the stem sap flow was reduced in the V-4CL-A transgenics and a moderate reduction was detected in the F-4CL-A transgenics (Figure 4A), indicating that the stem sap flow was altered in the transgenics. Then we examined the hydraulic conductance in stem. The hydraulic conductance was significantly decreased in the *V-4CL-A* transgenics but did not show changes in the *F-4CL-A* transgenics compared to the WT (Figure 4B). The distribution of water contents in stem was also analyzed by using a near-infrared imaging system (Supplementary Figure S2). The stem showed different abundance of water distribution. The percentage of the low abundance of water distribution area was significantly higher in the *V-4CL-A* transgenics than that in the *F-4CL-A* and WT plants (Figure 4C). Taken together, the results indicated that vessel-specific suppression of lignin biosynthesis affects stem water conductance, and therefore, the long distance transportation of water was impaired.

### Vessel-Specific Suppression of Lignin Biosynthesis Increased Susceptibility to Drought Stress

**FIGURE 5:**
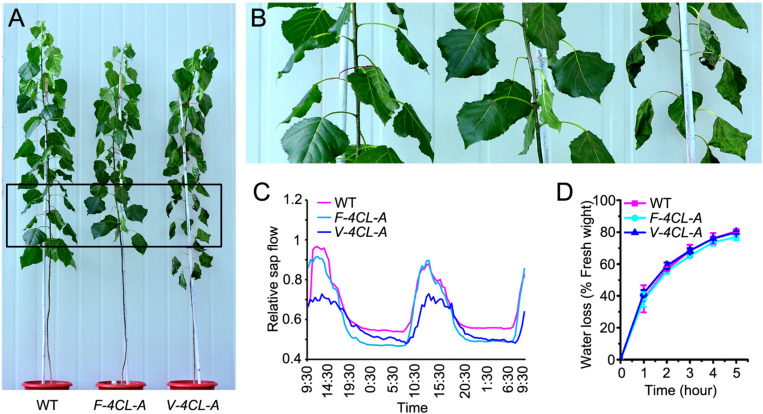
Vessel-specific downregulation of *4CL1* expression resulted in susceptibility to drought stress. **(A)** The transgenic response to drought condition. **(B)** Zoomed-in image of the leaf blade in rectangled area in **(A)**. **(C)** Measurement of the stem sap flow under drought condition and three biological replicates were carried out. One representative graph is shown. **(D)** Water loss rate of leaves in different transgenics. Water loss was calculated at different times after detaching from the tree at room temperature. The values represent means ± SE of 30 leaves from 6 clonally propagated plants from 3 independent lines.

To examine how the lignin-modified transgenics respond to drought stress, we carried out drought treatment for the *F-4CL-A*, *V-4CL-A* transgenics, and WT plants. After 10 days of drought treatment, the *V-4CL-A* transgenics were severely stressed, showing dehydrated and wilted leaves (Figures 5A,B), while the growth of WT and *F-4CL-A* transgenic plants displayed less stressed. Then, we measured the rate of leaf water loss and monitored the stem sap flow during drought treatment. The stem sap flow in the *V-4CL-A* transgenics was considerably lower in the daytime (Figure 5C) but showed no difference in the *F-4CL-A* transgenics compared to the WT under drought treatment. However, the water loss rate of leaf did not show significant difference between the transgenics and the WT (Figure 5D). Together, these results suggest that lignin modification in vessels resulted in more vulnerability to drought stress, likely due to inefficiency of long distance transportation of water through vessel elements.

### Vessel-Specific Suppression of Lignin Biosynthesis Affected Tree Growth in Field

**FIGURE 6:**
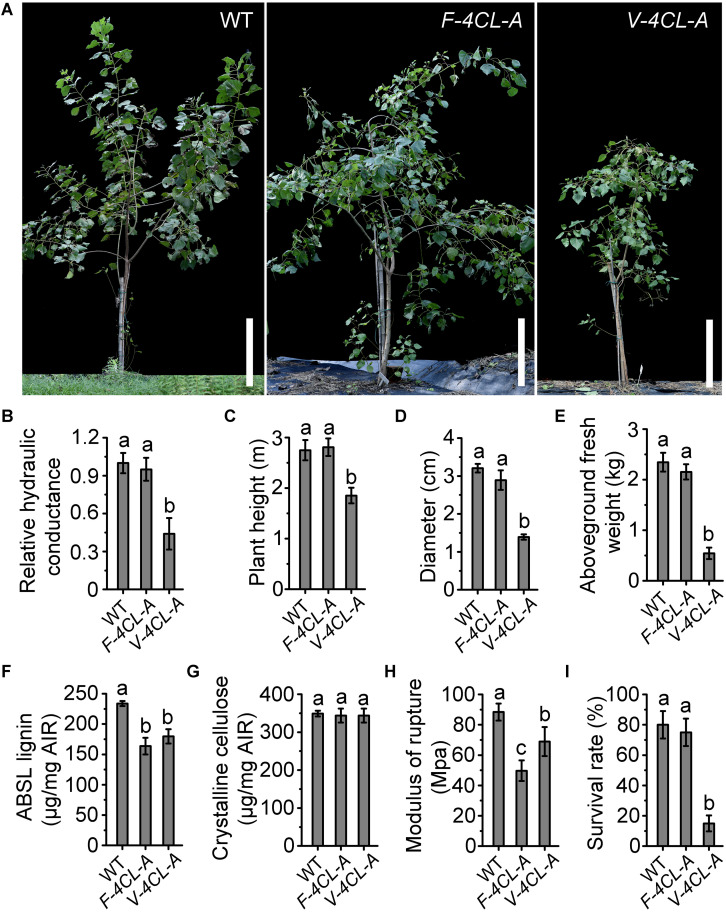
Field performance of the transgenic *Populus*. **(A)** The vessel-specific (*V-4CL-A*) and fiber-specific (*F-4CL-A*) downregulation of *4CL1* transgenics were grown in field for 1 year. Scale bar, 50 cm. **(B)** The hydraulic conductance in control plants was set as 1. Relative hydraulic conductance: means ± SE of six clonally propagated plants from three independent lines. **(C–E)** Plant height **(C)**, stem diameter **(D)**, and aboveground fresh weight **(E)**. Results are means ± SE of 12 clonally propagated plants from 3 independent lines. **(F,G)** Lignin content **(F)** and crystalline cellulose content **(G)** in the stem of the filed grown trees. Results are means ± SE of three biological replicates (lines). **(H)** Modulus of rupture (MOR) of the xylem tissue in stems. The values represent means ± SE of three biological replicates (lines). Different lowercase letters indicate significant differences at *p* < 0.01 by ANOVA. **(I)** The survival rate was calculated based on the initial planted trees (each line with 30 individuals) in field.

To examine how the lignin-modified transgenics perform in field, the transgenic trees were planted in field at our crop cultivation and breeding station and monitored for their growth performance in a full growth season (from November 2015 to November 2016) (Figure 6A). The *F-4CL-A* transgenics grew normally, similar to the control trees in height, stem diameter, and aboveground biomass (Figures 6A,C–E). The *V-4CL-A* transgenics displayed retarded growth with significant reduction in height, stem diameter, and aboveground biomass (Figures 6A,C–E). Plant height was reduced by 23–30%, stem diameter by 45–53%, and aboveground biomass by 65–80% in the *V-4CL-A* transgenics compared to the WT (Figures 6C–E). The field-grown *V-4CL-A* transgenics also showed deficient water transportation with 45–60% reduction in hydraulic conductance (Figure 6B). Both transgenics in field showed an appearance of slight drooping, which is likely caused by the softer stems and branches. The lignin content was reduced in both *V-4CL-A* and *F-4CL-A* transgenics, and the crystalline cellulose was not changed (Figures 6F,G). Lignin modification changed stem mechanical strength with much more reduction in the *F-4CL-A* transgenics than in the *V-4CL-A* transgenics (Figure 6H). The survival rate of the WT and the *F-4CL-A* transgenics was 80% after 1 year in field, but only 15% of the *V-4CL-A* transgenics survived (Figure 6I). These results demonstrated that lignin modification in xylem fiber has insignificant effect on plant growth, whereas the vessel-lignin modification results in substantial growth defects in field, which may be caused by deficiency of long distance transportation system in the stem.

## Discussion

The emergence of lignin is along with evolution of vascular plants and is thought to be critical for plant terrestrialization on the land (Weng and Chapple, 2010). However, how lignin plays a role in maintaining proper cell functionality related to plant growth is yet to be demonstrated. In this study, we modified lignin biosynthesis in a cell-specific manner through downregulation of *4CL1* expression in vessels and fibers, respectively. The lignin-modified *Populus* trees allow us to investigate the lignin physiological function in tree growth. Our results reveal that lignin biosynthesis in different types of cells has a distinct effect on plant growth.

In angiosperm trees, xylem is constituted with thick-walled vessel elements and fibers along with parenchyma cells. Lignin is a main composition of secondary cell walls in the thick-walled cells in xylem. Lignin biosynthesis in vessel elements and fibers of angiosperm xylem is distinctively regulated (Osakabe et al., 1999; Li et al., 2000, 2001; Yang et al., 2013; Smith et al., 2017; Zeng et al., 2017; De Meester et al., 2018; Gui et al., 2020). Studies have shown that deposition of lignin in an accurate temporospatial manner is crucial for plant growth and development (Zhong and Ye, 2007; Gui et al., 2011; Zhao and Dixon, 2011; Zhao et al., 2013). However, it is unclear how lignin biosynthesis affects the cell functionality in xylem. Our recent study indicated that vessel-specific and fiber-specific shutdown of lignin biosynthesis pathway in *Populus* showed different effect on the tree growth (Gui et al., 2020) suggesting that lignin biosynthesis in different cells has distinct effect on growth.

Lignin biosynthesis in vessels is closely related to plant growth. In Arabidopsis, vessel-specific reintroduction of the *C4H* and *CCR1* to the *c4h* and *ccr1* mutant, respectively, is able to recover their vessel integrity and partial restoration of their dwarfed phenotype (Yang et al., 2013; De Meester et al., 2018). However, it is unclear how vessel lignin causes defective growth. In this study, we analyzed the xylem system functionality in the transgenic *Populus*, which were modified lignin biosynthesis in vessel and fiber cells, respectively. Compared to the WT pants, stem sap flow and hydraulic conductance of xylem transportation system were significantly reduced in the *V-4CL-A* transgenics, but little changed in the *F-4CL-A* transgenics. Interestingly, the stomata conductance and water loss rate did not show difference between the *V-4CL-A* and *F-4CL-A* transgenics, indicating that the leaf transpiration was not modified in the transgenics. This suggests that the lower water content in the stem of *V-4CL-A* transgenics may be attributed to the deficiency of long distance transportation of water in the xylem system, not due to the leaf transpiration changes in the transgenics. Consistent with this, the *V-4CL-A* transgenics is more vulnerable to drought stress. The *V-4CL-A* transgenics with lignin modification in vessels displayed dwarfism, low survival rate, and reduction of aboveground biomass yield after 1 year of growth in field. Meanwhile, the *F-4CL-A* transgenics with lignin modification in fibers grew normally. Together, the study underscores the importance of lignin biosynthesis in vessels for long distance of water transportation, which is crucial for plant growth and survival, especially under the drought stress condition. On the other hand, *V-4CL-A* and *F-4CL-A* transgenics in the field showed the appearance of slight drooping, while the stem mechanical strength was much more significantly reduced in the *F-4CL-A* transgenics. Studies have showed lignin biosynthesis effect on the stem mechanical strength (Li et al., 2003; Voelker et al., 2010; Gui et al., 2020); the data in this study indicate that, particularly in fiber cells, lignin biosynthesis plays a primary role in reinforcing the mechanical strength.

Abundant on the globe, cell wall biomass has tremendous potential use for production of renewable energy and biomaterials. For a long time, it is highly attended for improvement of cell wall biomass utilization through modification of lignin biosynthesis. However, modification of lignin biosynthesis in plant is often associated with growth defects (Li et al., 2010; Voelker et al., 2010; Bonawitz and Chapple, 2013; Gui et al., 2019). Fiber-specific modification of lignin biosynthesis showed little penalty on growth, although the growth performance needs to be further examined in field for a longer period of time. Nevertheless, the findings yielded from the current study provide insightful information to elucidate how lignin biosynthesis affects growth, which in turn could aid in the development of new strategy for modification of wood biomass without growth penalty.

## Materials and Methods

### Plant Materials and Growth Conditions

*Populus deltoides* × *P*. *euramericana* cv. “Nanlin895” was used in this study. The transgenic trees were grown in a phytotron under conditions of 60% relative humidity, 12-h photoperiod at 9,000 lux, and at 23°C for 2 months and then moved to a greenhouse for phenotypic and physiological characterization. In addition, the 2-month-old trees were transplanted in a field (Crop cultivation and breeding station, Shanghai Institute of Plant Physiology and Ecology, 39.937128° N, 121.122304° E) for field analysis.

### Vector Construction

About 2 kb of the *WND1B* (*Potri.001G448400*) promoter region were cloned from *Populus* (Zhao et al., 2014) and fused with 35S mini promoter (regions from −90 to +8) (Benfey and Chua, 1990) and then subcloned into a *pCAMBIA2300-GUS* vector upstream of uidA (*GUS*). For cell type-specific suppression of *4CL1* (*Potri.001G036900*) gene in *Populus*, the *4CL1* cDNA was cloned from *Populus deltoides* × *P*. *euramericana* cv. “Nanlin895” and inserted into the vector *pCAMBIA2300:WND1Bp-35S mini-GUS* and *pCAMBIA2300:XCP1p-35S mini-GUS* in antisense orientation to obtain the vector construct *pCAMBIA2300:WND1Bp-35S mini-4CL1AS* (*F-4CL-A*) and *pCAMBIA2300:XCP1p-35S mini-4CL1AS* (*V-4CL-A*), respectively. The vector construct was transferred into the *Agrobacterium* strain GV3101 for genetic transformation according to the procedure (Li et al., 2003). Primers used in this study are listed in Supplementary Table S1.

### Morphological Analysis

Transgenics with suppression of *4CL* in fibers (*F-4CL-A*) and in vessels (*V-4CL-A*) were generated. 43 independent lines of *F-4CL-A* and 46 independent lines of *V-4CL-A* transgenics were identified, respectively. Of them, three to five transgenic lines were clonally propagated through micro-cutting for detail characterization. Morphological parameters, including plant height, internode length, stem diameter, leaf blade length, and leaf blade width, were measured in 2-month-old trees grown in phytotron or measured in 1.5-year-old trees, which were grown in field condition. Plant height was determined as the distance between shoot tip and stem base; stem diameter was measured 5 cm up from the stem base; internode length, leaf blade length and width were measured from the 11th to 20th internodes from shoot top. Aboveground, part was cut for fresh weight measurement.

### Gene Expression Analysis

Total RNA was extracted from the stem of *Populus* at 2-month-old stage using a total RNA kit following the manufacturer’s instructions (Omega). Transcript level of *Pd4CL1* was determined as described previously using a QuantStudio 3 Real-Time PCR Systems (Thermo Fisher Scientific). Gene expression data were normalized using *Populus actin2* gene (*Potri.001G309500*) as a reference. Primers used in this study are listed in Supplementary Table S1.

### Stem Hydraulic Conductance Measurement

Stem hydraulic conductance was measured with a High Pressure Flow Meter (HPFM, Dynamax) following the manufacturer’s instructions. In brief, the aerial part of the 2-month-old trees was collected and quickly connected with an HPFM instrument adaptor (HPFM, Dynamax). The conductivity of water flow was measured using a quasi-steady-state flow model meter. To minimize potential impact of the diurnal cycle on hydraulic conductance, the measurements were carried out between 10:00 a.m. and 3:00 p.m. in the phytotron under 60% relative humidity at 23°C).

### Sap Flow Measurement

The PTM-48A Monitor combined with an SF-5P sensor was used for automatic monitoring of the sap flow in the plant stem following the manufacturer’s instructions (Phyto-Sensor Group). The SF-5P sensor was mounted on the stem at 20 cm above the stem base for continuous recording for 3 days. Three sensors were used for each measurement at the same time.

### Measurement of Water Content Distribution in Stem

A phenotyping system LemnaTec 3D Scanalyzer (LemnaTec, GmbH, Wuerselen, Germany), which is equipped with visible (VIS) and near-infrared (NIR) image acquisition, was used for growth phenotyping and analysis of water content distribution in the stem. Two types of images were acquired. The visible images were recorded from top (exposure 25, gain 370, zoom 1,500, focus 3,200, iris 1,500), and from the side (exposure 16, gain 370, zoom 100, focus 3,000). The NIR images were recorded from the top (exposure 30,030, gain 100, zoom 1, focus 1, iris 2,500) and from the side (exposure 30,030, gain 100, zoom 1, focus 1,112, iris 3,200). The acquired images were processed by LemnaGrid, and then the phenotype and water content distribution data were calculated using LemnaMiner.

### Water Loss Analysis

Water loss rate in leaf was evaluated. Fully developed leaves at the same internodes from different types of transgenics were detached and weighed at different time intervals at room temperature. Water loss was calculated based on the initial leaf weight.

### Stomatal Conductance Measurement

Stomatal conductance was measured using a GFS-3000 portable photosynthesis system (Walz, Germany) under the condition of 350 μmol (CO_2_) mol^–1^, photosynthetic photon flux density of 1,200 μmol m^–1^ s^–1^, and air temperature of the leaf chamber at 25°C. Six leaves from each plant were measured.

### Drought Treatment

Trees at 6 months old were subjected to drought treatment without watering for 7–12 days and measured for stem sap flow. The drought condition was monitored using a wet sensor system (WET-2, Dynamax) to measure soil water content.

### Histochemical Staining and Immunolocalization

Phloroglucinol-HCl staining was performed as previously described (Gui et al., 2011). In brief, sections were stained with 1% phloroglucinol (w/v) in 12% HCl and observed with a microscope (Olympus BX53). GUS staining was performed as described (Gui et al., 2014). Stem internodes of *WND1Bp-35Smini-GUS* transgenics were incubated with a staining solution [100 mM NaPO_4_ (pH 7.0), 10 mM EDTA, 2 mM 5-bromo-4-chloro-3-indolyl-β-GlcA, 0.5 mM K_4_Fe(CN)_6_, 0.5 mM K_3_Fe(CN)_6_, 0.2% Triton X-100, and 20% methanol] at 37°C for 2 h. The samples were paraffin embedded and sectioned for observation. For immunolocalization, samples were fixed with acetone, paraffin-embedded and immunolocalized with GUS-specific antibodies as described previously (Gui et al., 2014).

### Lignin and Cellulose Analyses

Debarked stem from 2-month-old trees was collected and dried at 65°C. After grounding to fine powder by ball-milling, the sample was used to prepare alcohol-insoluble residues (AIR) according to Song et al. (2016). Acetyl bromide-soluble lignin (ABSL) and crystalline cellulose content were analyzed as previously described (Foster et al., 2010a,b).

### Mechanical Strength Analysis

Fresh xylem tissues in the stem of 2-month-old *Populus* trees grown in a glasshouse were prepared for mechanical strength analysis. Mechanical properties of xylem tissues were measured by a three-point bending test using a mechanical testing machine (HY-0580)^1^, and modulus of rupture (MOR) was calculated according to Kern et al. (2005).

## Data Availability Statement

All datasets generated for this study are included in the article/Supplementary Material, further inquiries can be directed to the corresponding authors.

## Author Contributions

SC, CH, JG, and LLi designed the research. SC, CH, LLu, SZ, YZ, and JS performed the research. JG, SC, and LLi analyzed the data and wrote the manuscript. All authors contributed to the article and approved the submitted version.

## Conflict of Interest

The authors declare that the research was conducted in the absence of any commercial or financial relationships that could be construed as a potential conflict of interest.

## Footnotes

1 http://www.hengyiyiqi.com
