# Supplementary material for: Cell-Specific Suppression of 4-Coumarate-CoA Ligase Gene Reveals Differential Effect of Lignin on Cell Physiological Function in Populus
Source: Front Plant Sci. 2020 Nov 17;11:589729. doi: 10.3389/fpls.2020.589729 (PMC7705072; doi:10.3389/fpls.2020.589729)
Supplement: Supplementary file 1 [file Table_1.DOCX]

**Supplemental Figures and Table:**

**Figure S1. Identification of fiber-specific promoters in *Populus*.**

**
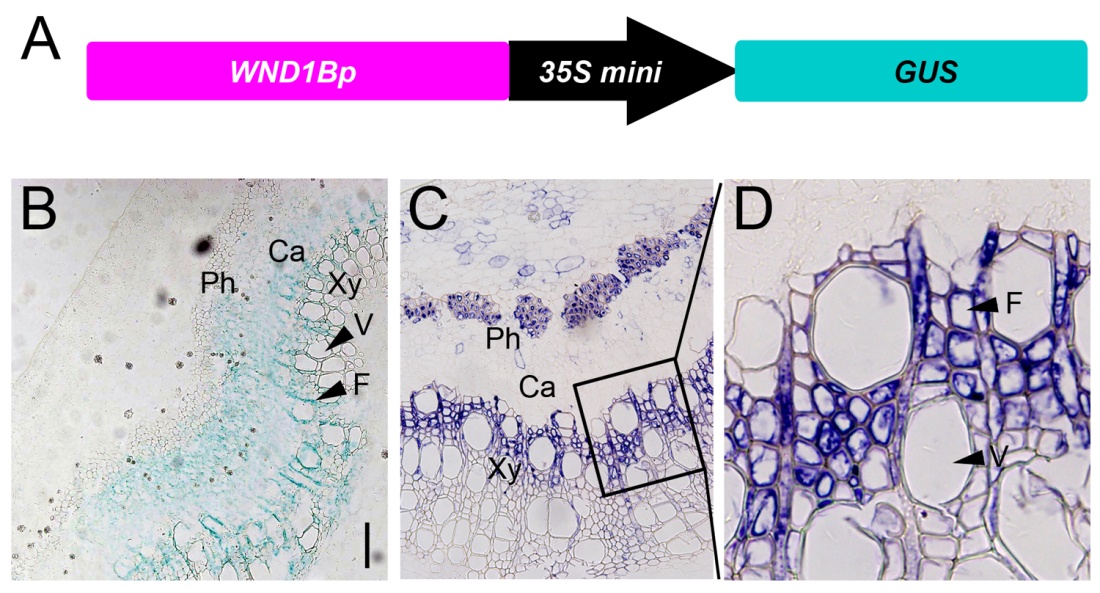
**

**(A)** Schematic diagram of the expression cassette designed to test the fiber-specific expression of *WND1B* chimeric promoter. *WND1Bp*, *WND1B* promoter; *35S mini*, *35S mini* promoter; *GUS*, β-glucuronidase reporter gene.

**(B)** Histochemical localization of WND1Bp-35Smini-GUS activity in stem of the transgenic *Populus*. Scale bar, 100 μm.

**(C, D)** Immunolocalization of GUS protein by GUS-specific antibodies in the *WND1Bp-35Smini-GUS* transgenic *Populus*. Scale bar, 50 μm.

Ca, cambium; Ph, Phloem; Xy, Xylem; F, fiber cell; V, vessel element.

**
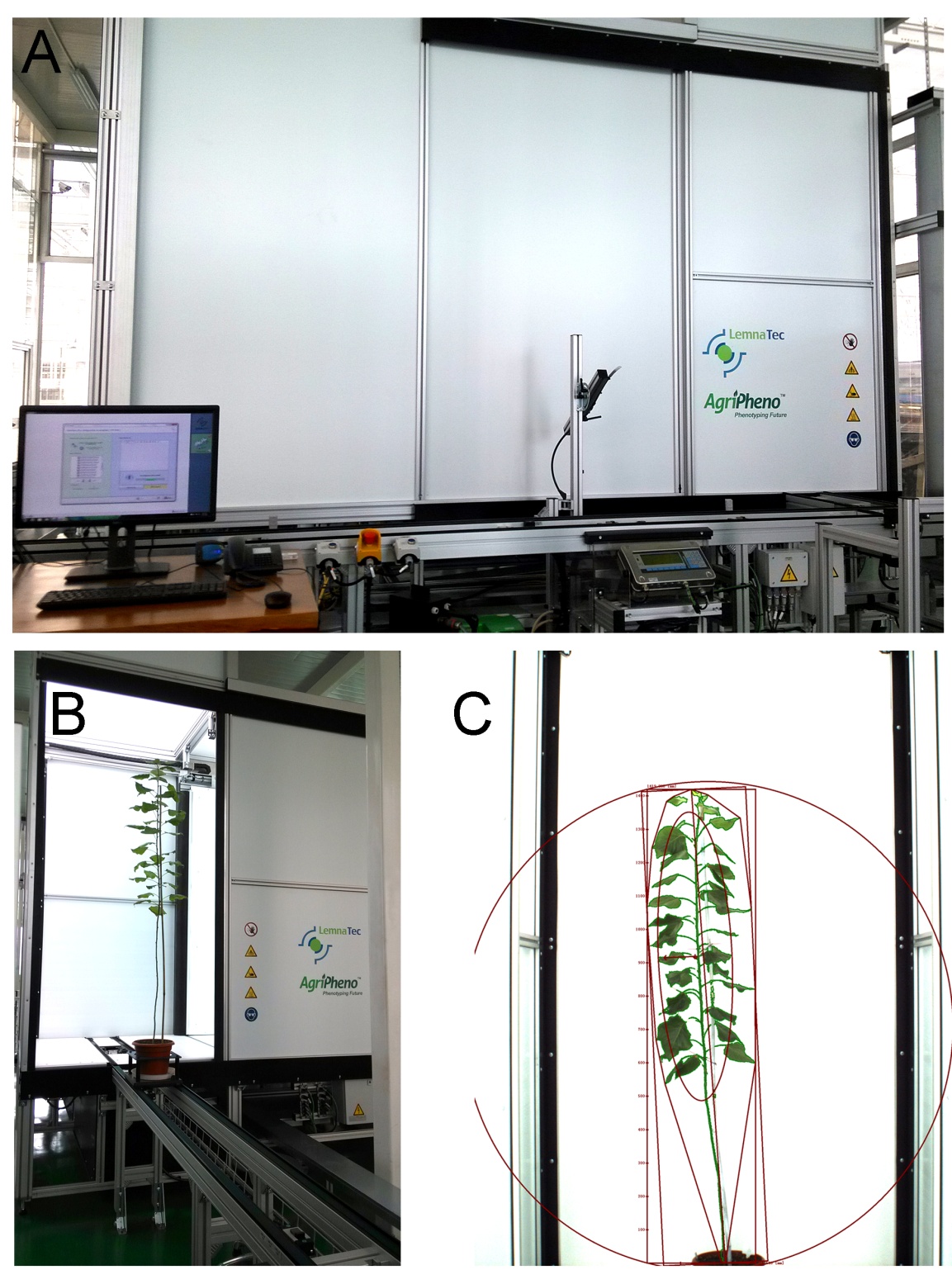
**

**Figure S2. Phenotyping system (Scanalyzer^3D^, Germany) was equipped with visible (VIS) and near-infrared (NIR) image acquisition for analysis of growth and water content distribution.**

**(A)** High-throughput phenotypic platform (AgriPheno, German).

**(B)** Plant loading into the Scanalyzer^3D^.

**(C)** Visible and near infrared chemometric imaging and analysis.

**
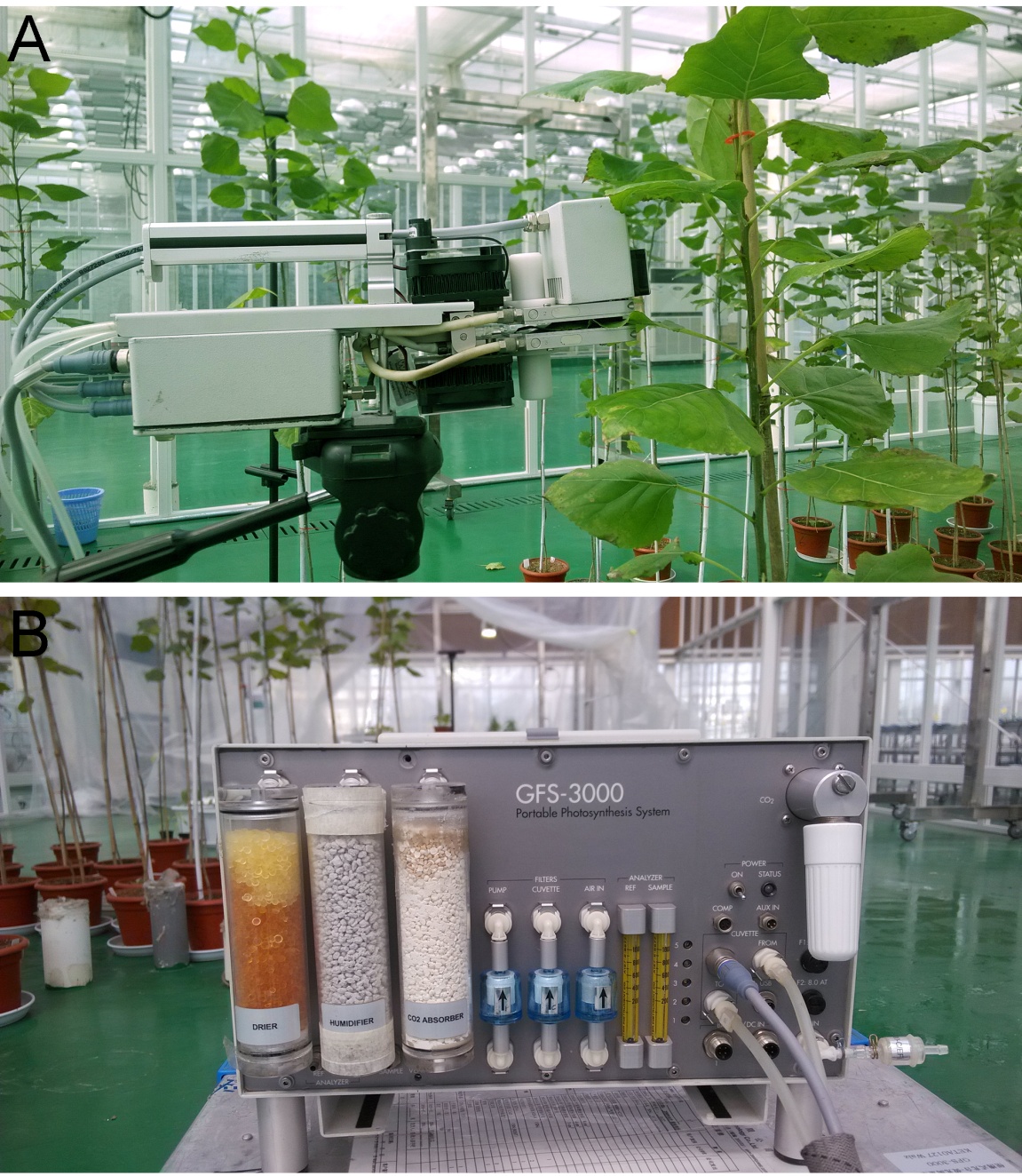
**

**Figure S3.** **GFS-3000 photosynthesis system was used to measure** **stomatal conductance.**

(A) Measurement of stomatal conductance with tripod mounted standard measuring head 3010-S.

(B) The GFS-3000 portable photosynthesis system.

**
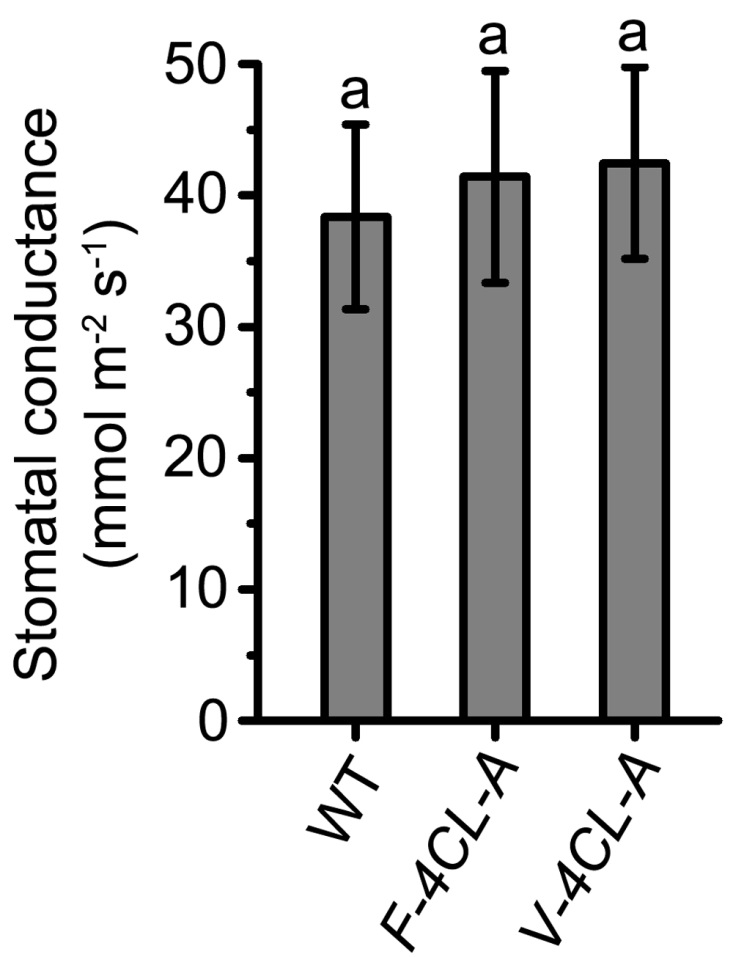
**

**Figure S4. Stomatal conductance of the transgenics with vessel-specific and fiber-specific down-regulation of *4CL1* expression.**

Stomatal conductance was measured in mature leave. Results are means ± SE of 10 clonally propagated plants.

**
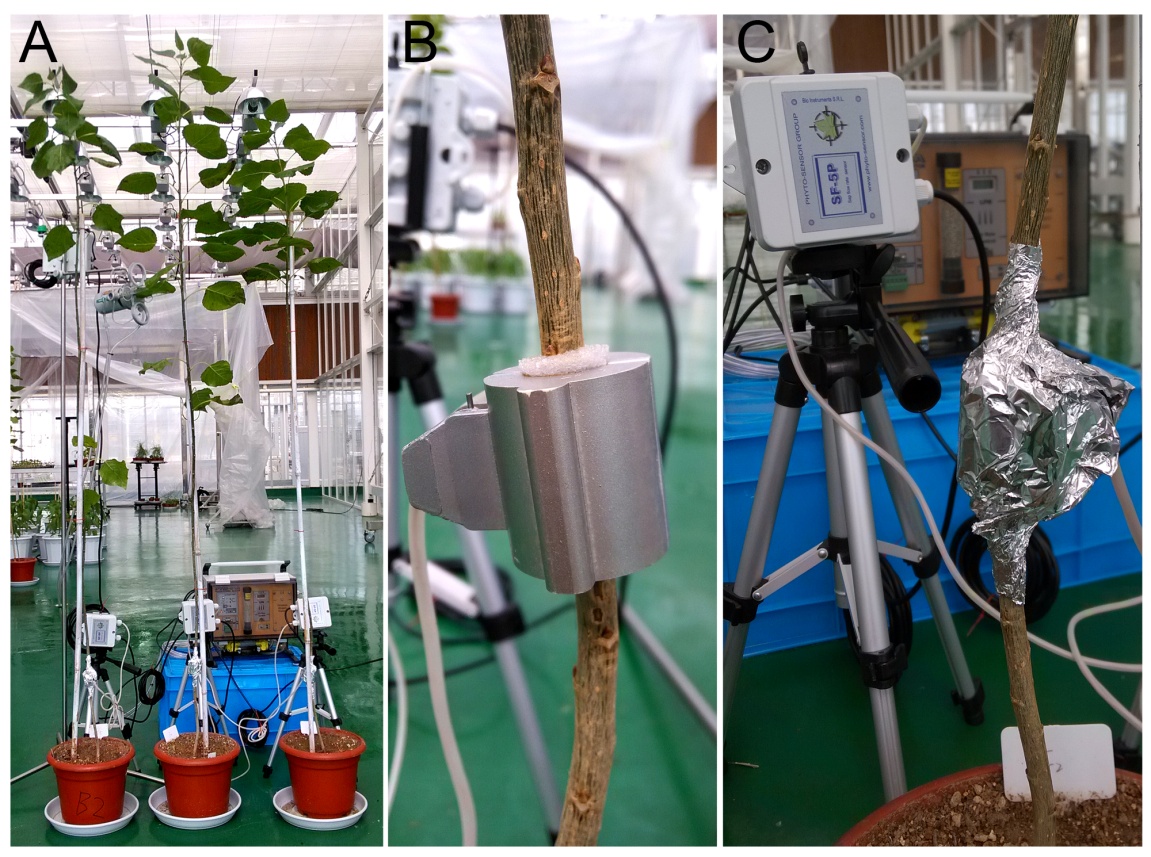
**

**Figure S5. Continuous monitoring of sap flow rate in plant stem in a glasshouse.**

**(A)** The PTM-48A Monitor combined with the SF-5P sensor was used for automatic monitoring relative variations of sap flow rate in stem of intact plants for 2-3 days.

**(B)** The SF-5P sensor was fixed at the stem of each plant about 20 cm up from the stem base.

**(C)** The SF-5P sensor module was fixed and connected to the PTM-48A Monitor.

**Supplemental Table 1. List of primers used in this study.**

| **Primer name** | **Primer sequence (5'-3')** | **Description** |
| --- | --- | --- |
| WND1Bp F | AACTACAGTAAAGAATTCCATTGAAAA | WND1B promoter cloning |
| WND1Bp R | TATATCTAGAATGGCTTGAAAATATATG |  |
| anti-4CL1 F | CTTTCTCCACCCCTGCAG | *F-4CL-A /* V*-4CL-A* vectors |
| anti-4CL1 R | TTAGGTACCTGGCAACGTTTC |  |
| 4CL1 F | CTAAGTTTCCTCAGGCCAGAC | qRT-PCR |
| 4CL1 R | TGCAGATCTCACCAGGCTG |  |
| ACT2 F | AAACTGTAATGGTCCTCCCTCCG | qRT-PCR |
| ACT2 R | GCATCATCACAATCACTCTCCGA |  |
